# Correction: A latent outcome variable approach for Mendelian randomization using the stochastic expectation maximization algorithm

**DOI:** 10.1007/s00439-026-02884-9

**Published:** 2026-11-12

**Authors:** Lamessa Dube Amente, Natalie T. Mills, Thuc Duy Le, Elina Hyppönen, S. Hong Lee

**Affiliations:** 1 Australian Centre for Precision Health, University of South Australia, Adelaide, SA 5000 Australia; 2 UniSA Allied Health and Human Performance, University of South Australia, Adelaide, SA 5000 Australia; 3 South Australian Health and Medical Research Institute, Adelaide, SA 5000 Australia; 4 Epidemiology Department, Jimma University, 378 Jimma, Ethiopia; 5 Discipline of Psychiatry, University of Adelaide, Adelaide, South Australia 5000 Australia; 6 UniSA STEM, University of South Australia, Mawson Lakes, SA 5095 Australia; 7 UniSA Clinical and Health Sciences, University of South Australia, Adelaide, SA 5000 Australia

**Correction: Human Genetics (2025)** https://doi.org/10.1007/s00439-025-02739-9

Following publication of our article, we identified a typographical error in the equation describing the update of the causal effect parameter in the Maximization (M) step of the stochastic expectation–maximization algorithm.

The published equation has been corrected as follows.

Published equation (incorrect):

$$\:{\tau\:}_{IVW}=\frac{{\sum}_{j}{I}_{j}{\widehat{u}}_{j}{\widehat{b}}_{j}se{\left({\mathrm{u}}_{j}\right)}^{-2}}{{\sum\:}_{j}{I}_{j}{\widehat{b}}_{j}^{2}se{\left({\mathrm{u}}_{j}\right)}^{-2}}$$

Correct equation:

$$\:{\tau\:}_{IVW}=\frac{{\sum}_{j}{I}_{j}{\widehat{\mathrm{g}}}_{j}{\widehat{b}}_{j}se{\left({\mathrm{g}}_{j}\right)}^{-2}}{{\sum\:}_{j}{I}_{j}{\widehat{b}}_{j}^{2}se{\left({\mathrm{g}}_{j}\right)}^{-2}}$$

